# Extracellular Vesicles of the Probiotic *Escherichia coli* Nissle 1917 Reduce PepT1 Levels in IL-1β-Treated Caco-2 Cells via Upregulation of miR-193a-3p

**DOI:** 10.3390/nu16162719

**Published:** 2024-08-15

**Authors:** Yenifer Olivo-Martínez, Sergio Martínez-Ruiz, Cecilia Cordero, Josefa Badia, Laura Baldoma

**Affiliations:** 1 Departament de Bioquímica i Fisiologia, Facultat de Farmàcia i Ciències de l’Alimentació, Universitat de Barcelona, 08028 Barcelona, Spain; yeni_olivo@hotmail.com (Y.O.-M.); sergio_martinez_ruiz@ub.edu (S.M.-R.);; 2 Biochemistry and Diseases Research Group, Facultad de Medicina, Universidad de Cartagena, Cartagena 130015, Colombia; 3 Institut de Biomedicina de la Universitat de Barcelona(IBUB), 08028 Barcelona, Spain; 4 Institut de Recerca Sant Joan de Déu (IRSJD), 08950 Barcelona, Spain

**Keywords:** gut microbiota, postbiotics, extracellular vesicles, inflammatory bowel disease, PepT1, miR-92b, miR-193a-3p

## Abstract

PepT1, a proton-coupled oligopeptide transporter, is crucial for intestinal homeostasis. It is mainly expressed in small intestine enterocytes, facilitating the absorption of di/tri-peptides from dietary proteins. In the colon, PepT1 expression is minimal to prevent excessive responses to proinflammatory peptides from the gut microbiota. However, increased colonic PepT1 is linked to chronic inflammatory diseases and colitis-associated cancer. Despite promising results from animal studies on the benefits of extracellular vesicles (EVs) from beneficial gut commensals in treating IBD, applying probiotic EVs as a postbiotic strategy in humans requires a thorough understanding of their mechanisms. Here, we investigate the potential of EVs of the probiotic Nissle 1917 (EcN) and the commensal EcoR12 in preventing altered PepT1 expression under inflammatory conditions, using an interleukin (IL)-1-induced inflammation model in Caco-2 cells. The effects are evaluated by analyzing the expression of PepT1 (mRNA and protein) and miR-193a-3p and miR-92b, which regulate, respectively, PepT1 mRNA translation and degradation. The influence of microbiota EVs on PepT1 expression is also analyzed in the presence of bacterial peptides that are natural substrates of colonic PepT1 to clarify how the regulatory mechanisms function under both physiological and pathological conditions. The main finding is that EcN EVs significantly decreases PepT1 protein via upregulation of miR-193a-3p. Importantly, this regulatory effect is strain-specific and only activates in cells exposed to IL-1β, suggesting that EcN EVs does not control PepT1 expression under basal conditions but can play a pivotal role in response to inflammation as a stressor. By this mechanism, EcN EVs may reduce inflammation in response to microbiota in chronic intestinal disorders by limiting the uptake of bacterial proinflammatory peptides.

## 1. Introduction

Inflammatory bowel disease (IBD) is a group of multifactorial and chronic inflammatory disorders that affect the gastrointestinal tract of genetically susceptible individuals. The two main forms of IBD are Crohn’s disease (CD) and ulcerative colitis (UC) [1,2]. Intestinal dysbiosis and altered immune responses to the gut microbiota are common traits in both IBD subtypes. The loss of immunological tolerance towards commensal bacteria in the intestine leads to a continuous activation of the intestinal immune system with the subsequent release of proinflammatory cytokines that promote tissue damage, thus exacerbating the inflammatory response.

Intestinal inflammation is a complex process that depends on multiple factors, including the dysregulation of specific transporters such as PepT1 (SLC15A1). PepT1 is a member of the proton-coupled oligopeptide transporter family that plays a relevant role in the maintenance of intestinal homeostasis. In the normal gut, PepT1 is primarily expressed in the enterocytes of the small intestine, where it mediates the absorption of di/tri-peptides derived from the digestion of dietary proteins. However, the expression of this transporter is minimal or practically undetectable in the colon [3,4]. Importantly, chronic inflammation leads to a significant increase in colonic PepT1 expression. Upregulation of PepT1 in colonic samples of IBD patients points to its role in the disease pathogenesis [1,5,6]. 

PepT1 exhibits a broad substrate specificity, able to transport structurally related peptide drugs such as β-lactam antibiotics, L-DOPA, and acetylcholine esterase inhibitors, among others [7,8]. In addition to its role in bioavailability of dietary peptides, PepT1 regulates the expression of specific miRNAs and regulates proteins along the crypt-villi axis of jejunum absorptive cells, contributing to important intestinal functions such as apoptosis and proliferation of intestinal epithelial cells [9]. Due to the essential role of the transporter, PepT1 expression and function are regulated by multiple mechanisms acting at different levels [10].

Regulation of colonic PepT1 expression occurs mainly at the transcriptional level in response to bacterial metabolites and derived peptides. In the colon, PepT1 mediates the uptake of bacterial peptides, including the proinflammatory peptide *N*-formyl-methionyl-leucyl-phenylalanine (fMLP) and peptidoglycan degradation products such as muramyl dipeptide (MDP) and L-Ala-γ-D-Glu-meso-diaminopimelic acid (Tri-DAP) [11]. The transport activity of PepT1 is closely related to the activation of intracellular immune receptors, in particular nucleotide-binding site–leucine-rich repeat (NOD) receptors, which recognize bacterial peptidoglycan fragments. NOD1 detects peptides containing meso-diaminopimelic acid (DAP) present in peptidoglycan of Gram-negative bacteria, while NOD2 senses MDP, which is common to all groups of bacteria. Interaction of NOD with the specific ligand initiates the signaling cascade that activates the NF-kB and mitogen-activated protein kinase (MAPK) pathways, leading to the subsequent expression of inflammatory genes. Therefore, the transport function of PepT1 plays a crucial role in determining the intracellular levels of NOD ligands and the consequent activation of inflammatory pathways [12,13,14,15]. In addition to peptide substrates, the expression of PepT1 is upregulated by proinflammatory cytokines IL-1β, IFN-γ, and TNF-α [5], as well as oxidative stress through the activation of the transcription factor nuclear factor erythroid 2-related factor 2 (Nrf2) [3]. Additionally, bacterial pathogens such as enteropathogenic *Escherichia coli* and *Citrobacter rodentium* can also upregulate PepT1 expression [5]. At the posttranscriptional level, mRNA degradation and translation are inhibited by miR-92b and miR-193a-3p, respectively [11,16]. Moreover, protein activity is regulated by phosphorylation through several kinases such as the AMP-kinase and also by interaction with other proteins involved in maintaining the proton gradient across the cell membrane (Na+/H+ exchanger, NHE3) or in the intracellular trafficking and proper location of PepT1 in the apical side of the enterocyte cell membrane [4,7].

Dysregulation of PepT1 expression has important implications in intestinal inflammation. Under pathophysiological conditions, bacteria present in the colon can generate significant amounts of MDP and Tri-DAP, which activate the expression of PepT1. The increased uptake of these peptidoglycan fragments triggers an inflammatory response in the intestinal epithelium through NOD downstream pathways [5]. In addition, uptake of fMLP by colonic epithelial activates the expression of major histocompatibility complex (MHC) class I molecules, making these cells more sensitive to bacterial peptides and prone to triggering an inflammatory response [2]. Indeed, treatment of Caco-2 cells with fMLP results in the activation of the proinflammatory transcription factors NF-κB and activator protein-1 (AP-1) [17]. Altered expression of miR-92b and miR-193a-3p has also been associated with PepT1 dysregulation and intestinal inflammation. The miR-92b inhibits PepT1 expression by binding to the 3′-unstranslated region of PepT1 mRNA and promoting its degradation. This finding points to miR-92b as a suppressor of proinflammatory responses induced by bacterial peptides in intestinal epithelial cells [11]. Moreover, an inverse correlation between PepT1 and miR-193a-3p levels has been reported in inflamed colon tissues with active UC. This miRNA inhibits PepT1 mRNA translation without affecting the mRNA levels. Consequently, miR-193a-3p reduces PepT1 protein levels and activity, thus suppressing the subsequent activation of inflammatory transcription factors [16]. Both miRNAs, miR92b and miR-193a-3p, are regulators of colonic PepT1 and contribute to maintaining intestinal homeostasis.

The relevant role of PepT1 overexpression on the inflammatory response against gut microbiota peptides opens new avenues for understanding and developing novel treatment strategies for IBD. Among microbiome-based strategies, manipulating the composition and function of the gut microbiota through probiotic interventions stands out. Currently, *Escherichia coli* Nissle 1917 (EcN) is among the probiotics approved for the management of gastrointestinal diseases, including UC. In addition, EcN is one of the best-studied probiotic strains due to its immunomodulatory and anti-inflammatory properties [18,19,20]. Despite the benefits and safety of probiotics, consuming viable bacteria may cause adverse events, particularly in patients with underlying medical conditions and weakened immune system. In these patients, probiotic therapy could aggravate inflammatory responses and convert the probiotic bacteria into harmful microbes. In this scenario, the newest microbiota-based therapies targeting the gut include the use of postbiotics [21]. According to the definition provided by International Scientific Association for Probiotics and Prebiotics (ISAPP), postbiotics are non-viable bacterial components and products produced by beneficial microbes that confer a health benefit on the host. This group includes bacterial cell fragments, biomolecules, and secreted bioactive compounds [22]. In this field, extracellular vesicles (EVs) released by probiotic and microbiota strains are emerging as potential new postbiotics [23,24,25].

Previous studies of our group have provided evidence on the role of microbiota EVs as crucial players in the interkingdom communication in the gut, acting as mediators of the microbiota functions on the host [26]. The EVs from the probiotic EcN and commensal *E. coli* strains regulate epithelial barrier integrity and function [27] and elicit regulatory mechanisms to preserve balanced anti- and pro-inflammatory responses in a strain-specific manner [28]. In intestinal epithelial cells, internalized EVs trigger an innate immune response through the activation of the NOD1 signaling pathway. Continuous stimulation of NOD1 by microbiota EVs leads to controlled inflammatory responses, essential for immune training and intestinal homeostasis [29]. In experimental models of IBD and rotavirus-induced diarrhea, oral administration of EVs from the probiotic EcN alleviates disease progression by counteracting the altered expression of inflammatory mediators and proteins involved in the epithelial barrier integrity and repair [30,31]. Moreover, in in vitro models of intestinal epithelial cells (goblet cells and enterocytes), EcN EVs modulate the expression of miRNAs dysregulated in IBD, particularly those that control the trefoil factor 3 (TFF3) and serotonergic genes [32,33]. In goblet cells, EcN EVs increased TFF3 expression by promoting downregulation of miR-7-5p through the phosphatidyl-inositol-3-kinase (PI3K) signaling pathway. By this mechanism, EcN EVs can improve barrier function and repair [32]. Also, in the IL-1β-induced inflammation model, EVs from this probiotic promoted downregulation of miR-24 and miR-200a, which indirectly control free serotonin levels by regulating the serotonin reuptake transporter SERT [33]. The comparison with EVs isolated from the commensal EcoR12 revealed that the regulatory effects were strain-specific.

This study aims to evaluate whether EVs isolated from the probiotic EcN and the commensal EcoR12 could prevent altered PepT1 expression under inflammatory conditions through the regulation of miR92b and miR-193a-3p. We used the interleukin (IL)-1β-induced inflammation model in Caco-2 cells. The effects of bacterial EVs were evaluated by analyzing the relative levels of PepT1 mRNA and the two regulatory miRNAs by RT-qPCR, as well as the PepT1 protein levels by ELISA and immunofluorescence confocal microscopy. The influence of microbiota EVs on PepT1 expression was also analyzed under conditions of normal induction by the natural transporter substrate Tri-DAP to elucidate the regulatory mechanisms functioning under both physiological and pathological conditions.

## 2. Materials and Methods

### 2.1. Bacterial Strains and Isolation of EVs

The probiotic *E. coli* Nissle 1917 (EcN) strain was obtained from Ardeypharm (GmbH, Herdecke, Germany). The commensal EcoR12 belongs to the ECOR reference collection. This *E. coli* strain was isolated from a stool sample of a healthy human adult [34].

Bacterial EVs were isolated as described previously [32]. Briefly, EcN and EcoR12 were grown overnight in Luria–Bertani broth. The bacterial cells were removed from the supernatants by centrifugation and subsequent sterile filtration (0.22 μm pore size filter, Merck, Millipore, MA, USA). The filtrate was concentrated using Centricon Plus-70 centrifugal filters 100 kDa cutoff (Merck, Millipore, MA, USA), and the EVs were pelleted by ultracentrifugation at 150,000× *g* for 1 h at 4 °C, washed and resuspended in phosphate-buffered saline (PBS). Sterility was confirmed by the absence of colonies on LB agar plates. Quantification of the protein concentration of EV samples was carried out with the Pierce BCA method. In addition, to assess equivalent EV amounts, samples were quantified using the lipophilic fluorescent dye FM4-64 (Thermo Fisher Scientific, Barcelona, Spain) as previously described [31]. This fluorescent dye intercalates into the vesicle membrane. EcN and EcoR12 EV samples with equal protein concentration (μg protein/mL) yielded comparable fluorescence intensity values. At 10 μg/mL, the mean fluorescence intensity values were 1.19 ± 0.04 for EcN EVs and 1.15 ± 0.03 for EcoR12 EVs. The integrity and homogeneity of the EVs from EcN and EcoR12 isolated following this protocol was verified by Cryo-Transmission Electron Microscopy (Cryo-TEM) performed as described previously [35] (Figure S1). Aliquots of the EVs were stored at −20 °C.

### 2.2. Cell Culture and Stimulation Conditions

Human colonic epithelial Caco-2 cells (ATCC HTB37) were grown in Dulbecco’s modified essential medium (DMEM), supplemented with 10% fetal bovine serum, 25 mM HEPES, 1% non-essential amino acids, and 1× penicillin–streptomycin (Corning, Fisher Scientific Inc., Barcelona, Spain, Catalogue # 30-002-CI). The cultures were maintained at 37 °C in a humidified incubator with 5% CO_2_. Routinely, cells were subcultured with trypsin-EDTA (Gibco-BRL, Fisher Scientific Inc., Barcelona, Spain) when they reached 80% confluency.

To analyze the regulation of PepT1 expression by microbiota EVs, two stimulation conditions were stablished in Caco-2 cells: (i) Polarized cell monolayers treated with IL-1β as a model of intestinal inflammation. (ii) Polarized cell monolayers exposed to Tri-DAP (L-Ala-γ-D-Glu-meso-diamino-pimelic acid) as a model of PepT1 regulation by substrate bacterial peptides.

The cellular model of intestinal inflammation used in this study was performed as previously described [32]. Briefly, Caco-2 cells were seeded (2 × 10^5^ cells/mL) in 12-well plates and cultured for 14 days. The medium was changed every two days. The cell monolayers were incubated with EcN EVs or EcoR12 EVs (60 μg/mL) for 3 h, and afterwards treated with IL-1β (10 ng/mL) for 48 h. In the Tri-DAP model, the experimental set up was the same, except that after incubation with EcN EVs or EcoR12 EVs (60 μg/mL, 3 h), the cell monolayers were treated with Tri-DAP at 5 μg/mL (InvivoGen, Ibian Technologies, Zaragoza, Spain) for 8 h. Stock solutions of IL-1β and Tri-DAP were prepared in PBS. During the experiments, the EVs, Tri-DAP, and IL-1β were maintained in the culture medium. In both models, non-treated cell monolayers incubated in the absence or presence of EVs were processed as control. Cells were collected for RNA extraction, and the culture supernatants were centrifuged (10,000× *g* for 20 min at 4 °C) and stored at −80 °C until analysis.

### 2.3. Cell Viability Assays

Cell viability was estimated by the MTT (3-(4,5-Dimethylthiazol-2-yl)-2,5-diphenyl tetrazolium bromide) assay as described previously [33]. The results were expressed as the percentage of cell survival relative to untreated control cells.

### 2.4. RNA Extraction and Quantitative Reverse Transcription-Polymerase Chain Reaction (RT-qPCR)

Total RNA was isolated from Caco-2 cells using the miRNeasy mini-Kit (Qiagen, Crawley, UK). Single-stranded cDNA was synthesized from 1 µg total using the High-Capacity cDNA Reverse Transcription kit (Applied Biosystems, Foster City, CA, USA). The reaction mixture (total volume 20 µL) was incubated according to the manufacturer’s instructions. Quantitative PCR reactions were carried out on a QuantstudioTM3 real-time PCR system (Applied Biosystems, Foster City, CA, USA) using SYBR^®^ Green PCR Master Mix (Applied Biosystems, Foster City, CA, USA) as previously described [32]. The primers for PepT1 were sense 5′-CGTGCACGTAGCACTGTCCAT-3′ and antisense 5′-GGCTTGATTCCTCCTGTACCA-3. The primers used to analyze the proinflammatory cytokines IL-8 and TNF-α were already published [33]. The housekeeping GAPDH gene was used as the reference gene (primers sense 5′-GAGTCAACGGATTTGGTCGT-3′ and antisense 5′- GACAAGCTTCCCGTTCTCAG-3′). A control reaction was carried out in the absence of RNA. The expression stability of the reference gene under the different experimental conditions was verified (Figure S2). The relative gene expression was calculated using the 2^−ΔΔCt^ formula. The data were presented as the fold change relative to the experimental control (untreated cells).

### 2.5. Quantification of Mature miRNA Expression

For microRNA expression analysis, extracted RNA (5 ng/μL) was reverse transcribed using the miRCURY LNA RT kit (Qiagen, Crawley, UK), followed by quantitative PCR using the miRCURY LNA PCR Assay (Qiagen) applying the PCR program described previously [32]. U6 snRNA was used as the reference gene for normalization. The expression stability of the reference gene under the different experimental conditions was verified (Figure S2).

The specific primers used were hsa-miR-193-a3p miRCURY LNA primer (Qiagen, Crawley, UK) (MiRBase accession # MIMAT0000459), hsa-miR-92b-3p miRCURY LNA primer (Qiagen, Crawley, UK) (MiRBase accession # MIMAT0003218), and the U6 snRNA control primer set (Qiagen, Crawley, UK). The relative gene expression was calculated using the 2^−ΔΔCt^ formula. The data were presented as the fold change relative to the experimental control (untreated cells).

### 2.6. Quantification of Cytokines by ELISA

The secreted levels of IL-8 and TNF-α were quantified in the culture supernatants using enzyme-linked immunosorbent assay (ELISA) sets (BD Biosciences, San Jose, CA, USA). The results were expressed as pg/mL.

### 2.7. Quantification of PepT1 by ELISA

Caco-2 cells were grown for 14 days as indicated in the previous section. Following the indicated treatments, adherent cells were rinsed in cold PBS, detached with trypsin and collected by centrifugation (1000× *g* for 5 min). The cells were resuspended in fresh lysis buffer (Cloud-Clone Corp., Katy, TX, USA, Catalogue # CC-IS007-1) at a final concentration of 10^7^ cells/mL and incubated until the solution was clarified. The cell lysate was then centrifuged at 1500× *g* for 10 min at 2–8 °C and stored at −20 °C until use. PepT1 levels were quantified by the enzyme-linked immunosorbent assay Kit for Hydrogen Ion/Peptide Transporter 1 (HPEPT1) (Cloud-Clone Corp., TX, USA, Catalogue # SEE429Hu), according to the manufacturer’s instructions. The results were expressed as ng/mL.

### 2.8. Immunofluorescence, Confocal Microscopy, and Image Analysis

Caco-2 cells were cultured in an 8-well chamber slide (µ-Slide 8-well glass bottom, Ibidi, Inycom, Zaragoza, Spain). After the indicated treatments, cells were rinsed, fixed, permeabilized, and blocked as previously described [32]. Immunostaining of PepT1 was performed using anti-hPepT1 Recombinant Rabbit Polyclonal Antibody (1:500, Catalogue # BS-10588R, Fisher Scientific, Inc., Barcelona, Spain) for 4 h at room temperature and Goat anti-Rabbit IgG (H + L) Superclonal™ Secondary Antibody, Alexa Fluor^®^ 488 conjugate (1:200, Catalogue # A27034, Fisher Scientific Inc., Barcelona, Spain) for 2 h at room temperature. Nuclei were labeled with DAPI (Sigma Aldrich, Chemical Co., St. Louis, MO, USA) at a final concentration of 0.125 µg/mL for 20 min.

Images were taken using a Zeiss LSM880 confocal microscope (Carl Zeiss Iberia S.L., Jena, Germany) equipped with a 63×/1.4 objective, an Argon laser, and a 405 nm laser diode available at the Advanced Optical Microscopy Unit from the Scientific and Technological Centers (CCiT-UB), University of Barcelona. Fluorescence was recorded at 488 nm (green, PepT1) and at 405 nm (blue, nuclei). Images were acquired with a voxel size of 0.13 µm × 0.13 µm × 0.37 µm (x, y, z, respectively). For each experimental condition and replicate (*n* = 3), 5 different fields of view with 50–100 cells each were acquired. Images were processed and analyzed using the Fiji software v1.53t [36]. To analyze the mean intensity of PepT1 staining, images were first filtered with a median filter (radius = 1) and then segmented using the Huang algorithm [37]. Measurements were performed on all focal planes of the original images masked with the previously obtained binary stacks.

### 2.9. Statistical Analysis

All data were obtained from three independent experiments, each conducted in triplicate. The GraphPad Prism 7.0 software (GraphPad Software, Inc., La Jolla, CA, USA) was used for statistical analysis and graph creation. Data were analyzed for normal distribution using the Shapiro–Wilk test. The one-way analysis of variance (ANOVA) followed by Tukey’s post-test was applied to determine differences between treatments. Differences were considered as statistically significant at *p* < 0.05. Data are expressed as the mean ± standard error (SEM).

## 3. Results

### 3.1. Modulation of PepT1 by EcN or EcoR12 EVs Under Conditions of IL-1β-Induced Inflammation

Overexpression of PepT1 in the colonic epithelium of patients with IBD provides an entry route for bacteria-derived peptides such as MDP, fMLP, and Tri-DAP, which activate NOD signaling cascades and contribute to chronic inflammation [13]. Here, we investigated the ability of EVs from the probiotic EcN or the commensal EcoR12 to modulate PepT1 expression in the IL-1β inflammation model. For this assay, polarized Caco-2 cells were pre-treated with EVs (60 µg/mL) for 3 h and then exposed to IL-1β (10 ng/mL) for additional 48 h. In parallel, modulation of PepT1 expression by EcN or EcoR12 EVs was evaluated in Caco-2 cell monolayers in the absence of IL-β treatment as a control of physiological conditions.

**Figure 1:**
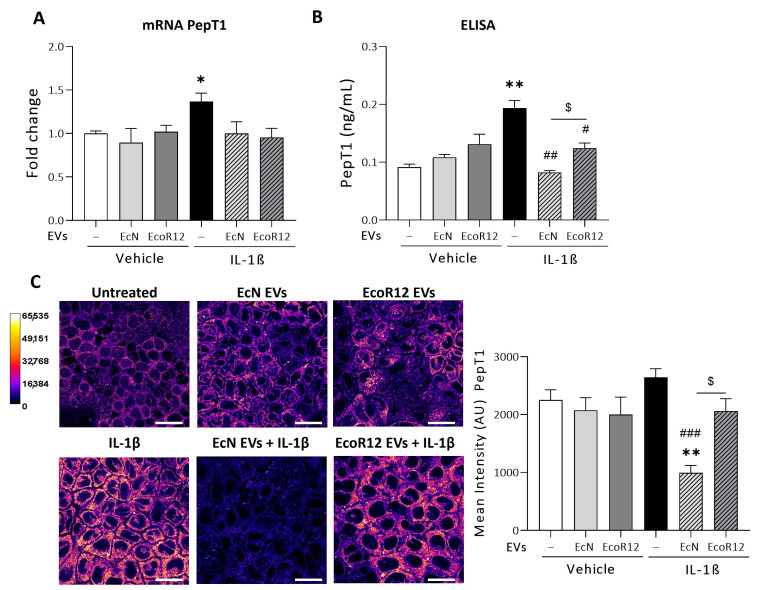
Regulation of PepT1 expression by EcN or EcoR12 EVs in the in vitro IL-1β-Inflammation model. Caco-2 cells were incubated with EVs (60 µg/mL) from EcN or EcoR12 for 3 h and then challenged with IL-1β (10 ng/mL) or the vehicle (PBS) for 48 h. In parallel, cells treated with EVs (60 µg/mL) from EcN or EcoR12 were incubated in the absence of IL-1β as a control. (**A**) Relative mRNA levels of PepT1 were assessed by RT-qPCR using GAPDH as the reference gene. Data are presented as mean ± SEM from 3 independent experiments. (**B**,**C**) Quantification of PepT1 protein levels by ELISA (**B**) and by immunofluorescence confocal microscopy (**C**). Representative confocal maximal projection images of cells treated as indicated are shown in Fire LUT after image processing. The calibration bar of Fire LUT intensity is shown in the left. Scale bar: 20 µm. Quantification of the PepT1 mean intensity is shown for each treatment on the right side. Data are given as mean ± SEM of arbitrary intensity units (AU) (*n* = 3 independent biological replicates). Statistical differences were assessed with one-way ANOVA, followed by post hoc Tukey’s. * *p* ≤ 0.05, ** *p* ≤ 0.01 vs. control untreated cells (white bars), # *p* ≤ 0.05, ## *p* ≤ 0.01, ### *p* ≤ 0.001 vs. IL-1β treated cells (black bars), $ *p* ≤ 0.05 between cells stimulated with EcN or EcoR12 EVs.

In the absence of inflammatory signals (conditions that mimic the intact intestinal epithelium), the PepT1 mRNA expression levels were not altered by treatment with EcN EVs or EcoR12 EVs compared to untreated control cells (Figure 1A). In the intestinal inflammation model, exposure to IL-1β significantly upregulated PepT1 expression (*p* ≤ 0.05). Incubation with EcN or EcoR12 EVs prevented the IL-1β-mediated upregulation of PepT1, resulting in mRNA levels similar to those of untreated control cells (Figure 1A). The expression of PepT1 was also analyzed at the protein level by ELISA and immunofluorescence confocal microscopy in Caco-2 cells under both established experimental conditions (Figure 1B,C).

In the model that mimics the intact intestinal epithelium, values of PepT1 protein were consistent with the mRNA expression levels assessed by RT-qPCR. Regardless of the methodology used for quantification, no significant differences in the PepT1 protein levels were detected between untreated or EV-treated cells (Figure 1B,C). However, no correlation between PepT1 mRNA and protein levels were observed in the IL-1β inflammation model, especially in cells treated with EcN EVs. As expected, IL-1β-treated cells exhibited higher PepT1 levels than untreated control cells (*p* ≤ 0.01). Remarkably, this increase in PepT1 protein was significantly counteracted by EcN EVs (*p* ≤ 0.01). EcoR12 EVs displayed a minor compensatory effect. Indeed, both PepT1 quantification methods yielded significant differences between cells treated with EVs from the probiotic or the commensal strain (Figure 1B,C).

### 3.2. Regulation of miRNAs That Target PepT1 mRNA by EcN and EcoR12 EVs under Conditions of IL-1β-Induced Inflammation

We next analyzed the ability of probiotic/microbiota EVs to modulate the expression of miRNAs known to regulate PepT1 mRNA by different mechanisms, particularly miR-193a-3p that inhibits PepT1 mRNA translation without altering the mRNA levels [16] and miR-92b that promotes PepT1 mRNA degradation [38].

**Figure 2:**
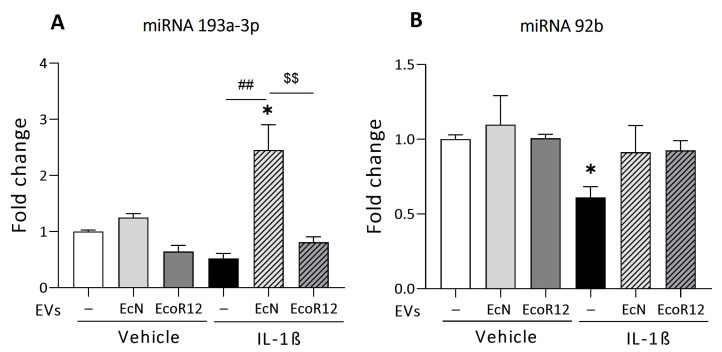
Regulation of miR-193a-3p (**A**) and miR-92b (**B**) by EcN or EcoR12 EVs in the IL-1β-Inflammation model. Caco-2 cells were incubated with EVs (60 µg/mL) from EcN or EcoR12 for 3 h and then challenged with IL-1β (10 ng/mL) or the vehicle (PBS) for 48 h. In parallel, cells treated with EVs (60 µg/mL) from EcN or EcoR12 were incubated in the absence of IL-1β as a control. Relative expression levels of the indicated miRNAs were quantified by RT-qPCR and normalized to the U6 reference gene. Data are expressed as mean ± SEM from three independent experiments. Differences were evaluated with one-way ANOVA, followed by post hoc Tukey’s. * *p* ≤ 0.05 vs. untreated control cells (white bars); ## *p* ≤ 0.01 vs. IL-1β treated cells (black bars), and $$ *p* ≤ 0.01 between cells stimulated with EcN or EcoR12 EVs.

In the intact intestinal epithelium model, the expression of both miRNAs did not significantly differ between untreated and EV-treated cells (Figure 2). In the inflammation model, treatment of Caco-2 cells with IL-1β resulted in the downregulation of miR-193a-3p (near significance, *p* = 0.056) and miR-92b (*p* ≤ 0.05) (Figure 2A). These results were consistent with the higher PepT1 mRNA and protein levels quantified in IL-1β-treated cells. Under these inflammatory conditions, the results showed differential regulation by EcN and EcoR12 EVs. The incubation with EcN EVs led to a nearly three-fold increase in the expression of miR-193a-3p compared to both untreated control cells (*p* ≤ 0.05) and cells treated with IL-1β in the absence of EVs (*p* ≤ 0.01) (Figure 2A). In contrast, incubation with EcoR12 EVs resulted in miR-193a-3p expression levels similar to those of untreated control cells. The overexpression of miR-193a-3p by EVs from the probiotic EcN under inflammatory conditions may account for the reduced PepT1 protein levels detected in this model. Concerning miR-92b, the results showed that EVs from both EcN and EcoR12 strains were able to prevent the IL-1β-mediated downregulation of miR-92b and preserve the basal expression levels of the untreated control cells (Figure 2B).

### 3.3. Effects of EcN and EcoR12 EVs on the Regulation of Tri-DAP Expression by Peptide Substrates

**Figure 3:**
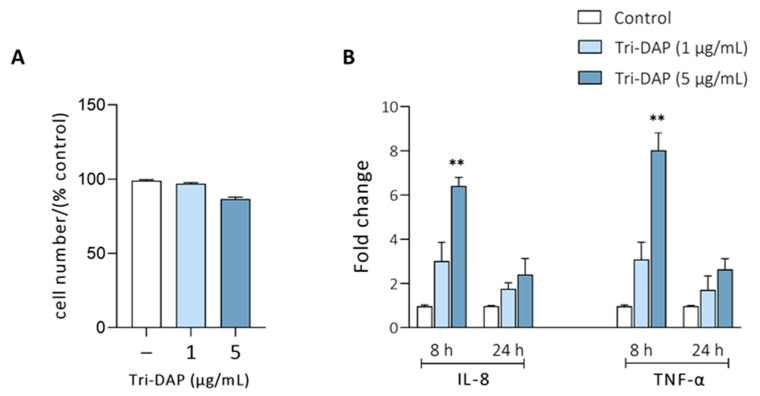
Setting up the experimental conditions for the Tri-DAP model. (**A**) Effect of the Tri-DAP concentration on cell viability assessed by the MTT assay. Caco-2 cell monolayers were exposed to Tri-DAP at the final concentration of 1 or 5 μg/mL for 24 h. (**B**) Influence of the Tri-DAP concentration on the expression of genes encoding the pro-inflammatory cytokines IL-8 and TNF-α. Caco-2 cells were exposed to Tri-DAP at the indicated concentrations for 8 and 24 h. Untreated cells were incubated in parallel as a control (white bars). The relative mRNA levels of the indicated genes were determined by RT-qPCR using GAPDH as the reference gene. Data are expressed as mean ± SEM from three independent experiments. Differences were evaluated with one-way ANOVA, followed by post hoc Tukey’s. ** *p* ≤ 0.01 vs. untreated control cells.

In the colon, bacterial peptides derived from peptidoglycan degradation (MDP, Tri-DAP) are internalized by the PepT1 transporter [13]. Moreover, di/tripeptide substrates are inducers of PepT1 expression in intestinal epithelial cells [10]. In this scenario, we decided to analyze whether EVs from probiotic and commensal strains could influence the activation of PepT1 expression by natural substrates, such as Tri-DAP. Prior to the study, we stablished the experimental conditions to select the Tri-DAP dose and the optimal incubation time to achieve a response. To this end, Caco-2 cell monolayers were incubated with two different concentrations of Tri-DAP (1 or 5 μg/mL) for 8 and 24 h. The MTT assay revealed that Tri-DAP, at the concentrations used, did not cause any cytotoxic effect, as cell viability remained close to 100% after 24 h incubation (Figure 3A).

The cell response was assessed by measuring the relative expression of IL-8 and TNF-α by RT-qPCR (Figure 3B). These proinflammatory cytokines serve as markers of NOD1 activation by internalized Tri-DAP. The results indicated that the maximal expression of IL-8 and TNF-α was achieved at 8 h of incubation with Tri-DAP at 5 μg/mL, indicating that Tri-DAP induced a quick inflammatory response that decreased with time.

Based on the results, we stablished the experimental conditions as follows: Caco-2 cell monolayers were pre-incubated with EcN EVs or EcoR12 EVs (60 μg/mL) for 3 h, and afterwards treated with Tri-DAP (5 μg/mL) for 8 h. For comparison, cell monolayers incubated for 8 h with the same concentration of EcN EVs or EcoR12 EVs were used. 

**Figure 4:**
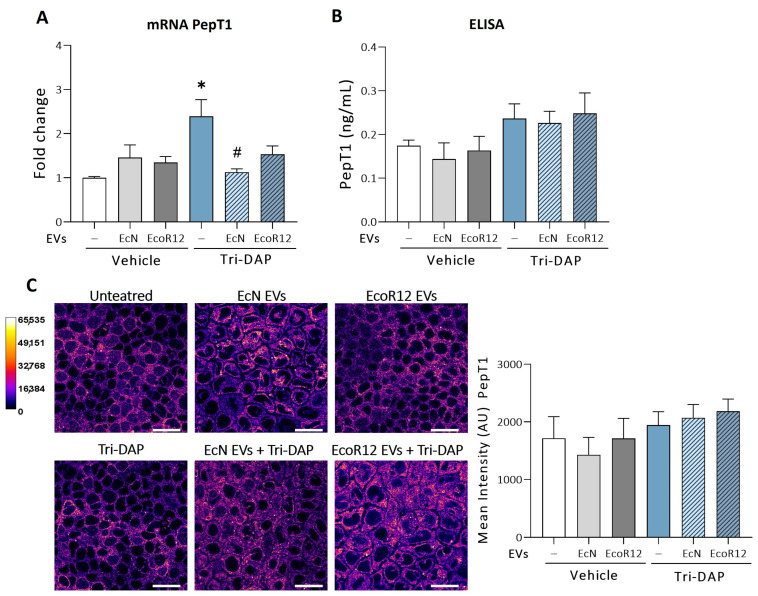
Regulation of PepT1 expression by EcN or EcoR12 EVs in the Tri-DAP induction model. Caco-2 cells were incubated with EVs (60 µg/mL) from EcN or EcoR12 for 3 h and then treated with Tri-DAP (5 µg/mL) or the vehicle (PBS) for 8 h. In parallel, Caco-2 cells treated with EVs (60 µg/mL) from EcN or EcoR12 were incubated in the absence of Tri-DAP as a control. (**A**) Relative mRNA levels of PepT1 were measured by RT-qPCR using GAPDH as the reference gene. Data are expressed as mean ± SEM from 3 independent experiments. (**B**,**C**) Quantification of PepT1 protein levels by ELISA (**B**) and by immunofluorescence confocal microscopy (**C**). Representative confocal maximal projection images of cells treated as indicated are shown in Fire LUT after image processing. The calibration bar of Fire LUT intensity is shown in the left. Scale bar: 20 µm. Quantification of the PepT1 mean intensity is shown for each treatment on the right side. Data are shown as mean ± SEM of arbitrary intensity units (AU) (n = 3 independent biological replicates). Statistical differences were assessed with one-way ANOVA, followed by post hoc Tukey’s. * *p* ≤ 0.05 vs. untreated control cells (white bars); # *p* ≤ 0.05 vs. Tri-DAP treated cells (blue bars).

In this model, the expression of PepT1 was assessed by RT-qPCR (Figure 4A). The relative PepT1 mRNA levels significantly increased in cells stimulated with Tri-DAP (*p* ≤ 0.05), a finding that was consistent with the activation effect of the transporter substrates. Remarkably, EVs from the probiotic EcN prevented Tri-DAP-mediated PepT1 upregulation (*p* ≤ 0.05). In these cells, PepT1 mRNA levels were comparable to those of untreated control cells. EVs from the commensal EcoR12 exhibited a minor protective effect, with differences that did not reach statistical significance (Figure 4A, Tri-DAP treatment). Comparable to the model of intact intestinal epithelium presented in Figure 1 (48 h incubation), treatment of Caco-2 cells with EcN or EcoR12 EVs for a shorter period did not affect PepT1 mRNA levels compared to untreated control cells (Figure 4A). Despite differences in the PepT1 mRNA levels, treatment with Tri-DAP did not result in higher PepT1 protein levels (Figure 4B,C). Indeed, quantification of PepT1 protein levels did not reveal significant differences between treatments (Tri-DAP and/or EVs).

**Figure 5:**
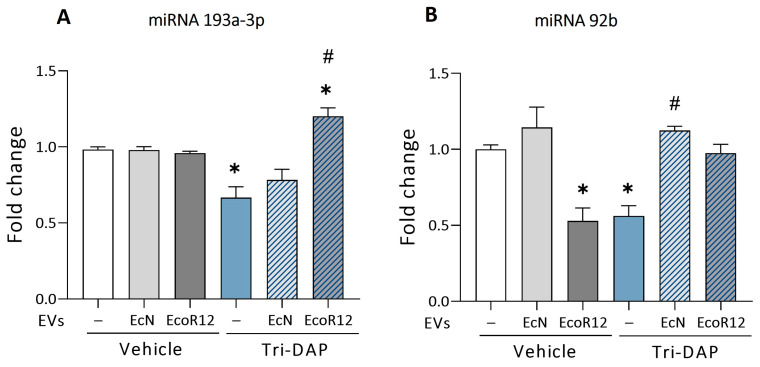
Regulation of miR-193a-3p (**A**) and miR-92b (**B**) by EcN or EcoR12 EVs in the Tri-DAP induction model. Caco-2 cells were incubated with EVs (60 µg/mL) from EcN or EcoR12 for 3 h and then treated with Tri-DAP (5 µg/mL) or the vehicle (PBS) for 8 h. In parallel, Caco-2 cells treated with EVs (60 µg/mL) from EcN or EcoR12 were incubated in the absence of Tri-DAP as a control. Relative expression levels of the indicated miRNAs were measured by RT-qPCR and normalized to the U6 reference gene. Data are expressed as mean ± SEM from three independent experiments. Differences were evaluated with one-way ANOVA, followed by post hoc Tukey’s. * *p* ≤ 0.05 vs. untreated control cells (white bars); # *p* ≤ 0.05 vs. Tri-DAP treated cells (blue bars).

The expression of miR-193a-3p and miR-92b was also analyzed in cells challenged with Tri-DAP (Figure 5). In the absence of probiotic/microbiota EVs, this pro-inflammatory bacterial peptide promoted significant downregulation of both miRNAs (*p* ≤ 0.05). The reduced expression of miR-92b correlated with the increased expression of the PepT1 mRNA target. Notably, the Tri-DAP downregulatory effect on miR-92b was counteracted by EVs from either EcN or EcoR12 strains. Again, EcN EVs showed a more prominent effect (*p* ≤ 0.05) (Figure 5B). In the absence of Tri-DAP, EVs from the commensal EcoR12 reduced miR-92b levels (*p* ≤ 0.05) (Figure 4C, control panel). This effect was not observed at longer incubation times (Figure 2).

Regarding miR-193a-3p, the expression profile differed depending on whether cells were treated with EcN or Ecor12 EVs. EcN EVs tended to preserve the basal expression levels of untreated control cells, whereas EcoR12 EVs upregulated miR-193a-3p over the basal expression level (*p* ≤ 0.05) (Figure 5A). In the absence of Tri-DAP, no differences were detected in miR-193a-3p levels between untreated or EV-treated cells (Figure 5A). Thus, in the absence of additional signals, EVs from EcN and EcoR12 did not modulate early or late miR-miR-193a-3p expression.

In the absence of Tri-DAP, the results of miRNA expression matched the expected protein expression profile. In the presence of Tri-DAP, the regulatory effects exerted by microbiota/probiotic EVs on PepT1 mRNA, miR-92b, and miR-193a-3p expression could explain why these cells exhibited similar PepT1 protein levels to untreated control cells. In this sense, although PepT1 mRNA levels were somehow higher in EcoR12 EVs + Tri-DAP-treated cells than control cells (Figure 4A), the upregulation of miR-193a-3p by EcoR12 EVs under these conditions could compensate for the expected higher synthesis of PepT1. In cells treated with EcN EVs + Tri-DAP, the relative expression of PepT1 mRNA, miR-92b, and miR-193a-3p did not significantly differ from those of untreated control cells, a fact that is consistent with similar PepT1 amounts detected. In the absence of EVs, the downregulation of miR-193a-3p by Tri-DAP should result in higher PepT1 protein levels. However, in cells stimulated with Tri-DAP, the total PepT1 amount did not differ from that of control cells (Figure 4B,C), suggesting that peptide substrates may activate additional regulatory mechanisms to preserve low levels of colonic PepT1 in order to maintain intestinal homeostasis.

### 3.4. Modulation of Proinflammatory Cytokines by EcN or EcoR12 EVs in Caco-2 Cells Stimulated with Tri-DAP

**Figure 6:**
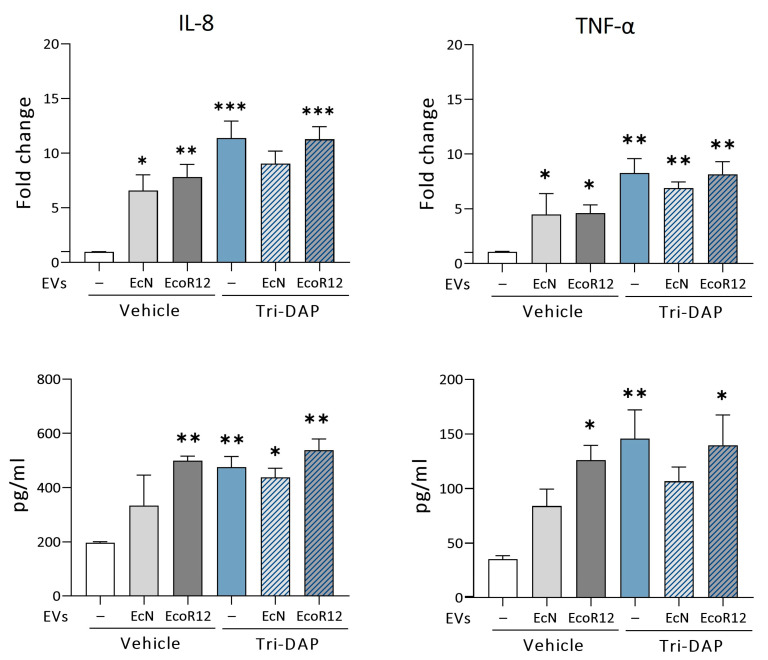
Expression analysis of proinflammatory cytokines in cells stimulated with Tri-DAP in the absence or presence of EcN Evs or EcoR12 Evs. Tri-DAP stimulation conditions: Caco-2 cells were incubated with Evs (60 µg/mL) from EcN or EcoR12 for 3 h and then treated with Tri-DAP (5 µg/mL) for 8 h (blue and dashed bars). Control conditions: Caco-2 cells were treated with Evs (60 µg/mL) from EcN or EcoR12 and incubated for 8 h (white and gray bars). Relative mRNA levels of the indicated cytokines were measured by RT-qPCR using GAPDH as the reference gene (upper panels), and secreted levels of IL-8 and TNF-α were quantified by ELISA in the culture supernatants. In all panels, data are presented as mean ± SEM from three independent experiments. Differences were evaluated with one-way ANOVA, followed by post hoc Tukey’s. * *p* ≤ 0.05, ** *p* ≤ 0.01, and *** *p* ≤ 0.001 vs. control cells.

The uptake of Tri-DAP by PepT1 and the following elevation of proinflammatory cytokines through the activation of NOD receptors are well documented [1]. In this context, we analyzed the ability of EcN or EcoR12 EVs to influence the expression of these inflammatory mediators after PepT1 activation. This analysis included cytokines IL-8 and TNF-α. Treatment of Caco-2 cells with EcN or EcoR12 EVs significantly induced IL-8 and TNF-α, both at the mRNA and protein levels, compared to untreated control cells (Figure 6). This finding was consistent with the activation of immune receptors (TLRs, NOD1) by microbial-associated molecular patterns (MAMPs) carried and delivered to epithelial cells by the bacterial EVs.

Treatment with Tri-DAP also triggered upregulation of IL-8 and TNF-α. In this case, the mRNA levels were even higher than those elicited by EcN or EcoR12 EVs. Notably, EVs from these intestinal *E. coli* strains did not modify the expression profile of the pro-inflammatory cytokines in response to Tri-DAP. The relative expression of mRNA and the levels of secreted IL-8 and TNF-α were similar between cells challenged with Tri-DAP both in the absence or presence of EVs. In cells incubated with EVs from the probiotic EcN, the expression of both proinflammatory cytokines tended to be lower. Although not statistically significant, these differences are compatible with the anti-inflammatory activity of EcN EVs previously reported [30,33].

## 4. Discussion

Dysregulation of PepT1 in the colon has been associated with chronic inflammatory diseases such as IBD and colitis-associated cancer [7]. In the colon, the mucosal surface is constantly exposed to microbiota and its derived bacterial metabolic products and proinflammatory peptides such as MDP, Tri-DAP, or fMLP. The poor expression of PepT1 in healthy colonic epithelial cells limits the uptake of bacterial peptides and the subsequent activation of inflammatory pathways. However, the normal PepT1 expression pattern is altered in IBD. Several factors may contribute to the enhanced expression of PepT1 in the colonic epithelium of IBD patients such as the high levels of proinflammatory cytokines [39,40,41] and dysregulation of miRNAs that target PepT1 mRNA [16,42]. In this clinical condition, overexpression of PepT1 amplifies inflammation by transporting bacterial proinflammatory peptides that activate NOD signaling cascades. This mechanism contributes to the abnormal immune response against the gut microbiota. In fact, in the experimental model of dextran sulfate sodium (DSS)-induced colitis, overexpression of PepT1 in the colonic mucosa of transgenic mice aggravated inflammation [13], whereas knockout (KO) PepT1 animals exhibited reduced levels of proinflammatory cytokines than wild-type animals. Interestingly, differences between KO-PepT1 and wild type mice were abolished by antibiotic treatment before colitis induction [43]. Moreover, there is evidence that overexpression of PepT1 regulates the colonic expression and secretion of certain miRNAs, such as miR-23, which is involved in active UC and CD, thereby contributing to disease progression [44]. All these findings point to PepT1 as a potential target for IBD treatment to ameliorate inflammation [6,45].

Previous studies of our group showed the efficacy of EVs from the probiotic EcN to improve inflammation and intestinal barrier disfunction in the experimental model of DSS-induced colitis in mice [30]. Further research allowed identification of the underlying molecular mechanisms, particularly those related with dysregulated miRNAs that control the trefoil factor 3 (TFF3) and serotonergic genes [32,33]. Based on these findings, here, we explored the ability of EcN EVs to modulate the expression of PepT1 and its regulatory miRNAs miR-193a-3p and miR-92b under inflammatory conditions using the well-characterized IL-1β inflammation model in Caco-2 cells [33]. The results showed that treatment with the proinflammatory cytokine IL-1β resulted in higher levels of PepT1 (both mRNA and protein) together with lower levels of both miRNAs compared to untreated control cells. Although in the absence of inflammation (control cells), EVs from the probiotic EcN regulate neither PepT1 expression nor its regulatory miRNAs; in IL-1β treated cells, EcN EVs significantly reduced the PepT1 protein amount to levels even lower than those of control cells. The main mechanism underlying this effect was the upregulation of miR-193a-3p, which inhibits PepT1 synthesis without altering the mRNA levels [16]. This is a strain-specific mechanism since EVs from the commensal EcoR12 did not upregulate miR-193a-3p under inflammatory conditions. Concerning miR-92b, EVs from both strains prevented the IL-1β-induced downregulation of miR-92b. The relative expression levels of this miRNA and its target PepT1 mRNA were undistinguishable from those of untreated control cells.

When analyzing the effects of EcN and EcoR12 EVs on the regulation of PepT1 by the natural bacterial substrate Tri-DAP, we found that EVs from both strains prevented Tri-DAP-mediated downregulation of miR-193a-3p and miR-92b and the associated increase in the PepT1 mRNA levels. However, in this model of PepT1 induction, EVs from the probiotic strain did not upregulate miR-193a-3p compared to untreated control cells. This suggests that EcN EVs did not control PepT1 expression under basal conditions but can play a pivotal role in response to inflammation as a stressor. Other regulatory mechanisms elicited by EcN EVs, such as the anti-inflammatory effects or regulation of genes and miRNAs that control intestinal serotonin levels, are only active under inflammatory conditions [33]. It should be noted that although Tri-DAP increases PepT1 mRNA levels, the total protein amount does not significantly differ from control or EV-treated cells. This suggests that PepT1 is tightly controlled in the healthy colonic epithelium at the protein level. The transcriptional activation of PepT1 induced by the abundant proinflammatory bacterial peptides in the colon may be compensated by post-transcriptional mechanisms other than miR-193a-3p to prevent strong proinflammatory responses. In this sense, a study aimed at elucidating the role of specific miRNAs that contribute to the posttranscriptional regulation of main drug transporters in the gut identified miRNAs whose expression negatively correlate with PepT1 protein levels. Besides miR-193a-3p, reporter gene assays confirmed the role of miR-193b-3p and miR-27a-3p in regulating PepT1protein levels [46].

It is important to consider that the gut microbiota plays a fundamental role in the training of the innate immune system, which should quickly recognize and mount inflammatory responses against pathogens. The uptake of Tri-DAP through PepT1 induced a pro-inflammatory response with increased production of IL-8 and TNF-α. This finding was consistent with the recognition of Tri-DAP by cytosolic NOD receptors and the subsequent activation of NF-κB [11]. The inflammatory response was not prevented by EcN or EcoR12 EVs. In fact, treatment of Caco-2 cells with EVs from both strains also triggered the expression and secretion of proinflammatory cytokines IL-8 and TNF-α. In this context, we previously showed that EVs released by commensal microbiota are internalized by intestinal epithelial cells trough endocytosis and interact with NOD1 at the endosomal compartment [47]. Since PepT1 expression is negligible in healthy colonic epithelia, endocytosis of microbiota EVs provide a mechanism for peptidoglycan transport into the host cell cytosol and the detection of microbial products that prime the innate immune system.

Clinical research is gradually moving towards personalized medicine. Understanding how host–microbe associations may impact human health and disease highlights microbiome research as a crucial element to drive future advancements in medicine and nutritional approaches [48,49]. This is especially relevant in the case of chronic intestinal inflammatory diseases, since current IBD therapies have substantial side effects, and in most cases their efficacy diminishes after long-term treatments [50]. Moreover, proper control of chronic inflammation may reduce long-term complications such as colorectal cancer [51]. Now, the discovery of novel probiotic strains for IBD treatment is a growing area. In this context, besides the well-known probiotic EcN, the gut colonizing bacteria *Akkermansia muciniphila* and *Faecalibacterium prausnitzii* are seen as promising candidates for next-generation probiotics [52,53]. The aim of biotherapies involving probiotics and prebiotics is to restore the gut microbiota composition to anti-inflammatory and mucosa-repairing profiles [54,55]. Considering that the beneficial effects of probiotics are mediated by bioactive bacterial molecules, the paradigm of microbiome-based interventions is currently shifting towards postbiotics [21,56]. The administration of postbiotics is a harmless strategy that exploits the biological activity of probiotic effectors, avoiding the risk of bacterial translocation in patients with disrupted intestinal barrier and altered immune responses. In addition, postbiotics generally exhibit good absorption and distribution, circumventing the technical challenges associated with bacterial viability and colonization efficiency. This characteristic facilitates their application in a wide variety of pharmaceuticals and functional foods [57,58].

Now, EVs derived from gut beneficial microbes are emerging as upcoming postbiotics [24,59,60,61]. In the gut, microbiota–host communication principally relies on metabolites, secreted biomolecules, and vesicles able to migrate through the mucus layer [62]. Accumulated evidence has shown that microbiota EVs serve as vehicles for the transport of functional bacterial molecules to host cells in a protected environment. Microbiota EVs enclose a wide variety of components produced by the parental strain, including typical MAMPs that engage host immune receptors, as well as specific proteins, metabolites, lipids, and nucleic acids that modulate host responses [26,63]. Therefore, some effects of the microbiota EVs are specific as they depend on the producer bacterial strain and cargo.

Despite intense research in animal models of human diseases demonstrating the benefits of EVs from certain beneficial gut commensals in ameliorating IBD through different mechanisms, mainly involving the reinforcement of the epithelial barrier and anti-inflammatory effects [30,64,65,66], translation of probiotic/microbiota EVs to human health as a postbiotic strategy requires deep knowledge of the action mechanisms. Concerning PepT1 expression, experimental models have been used to explore the ability of Lactobacillus strains to regulate PepT1 expression in the small intestine. Upregulation of PepT1 in the jejunum by *L. plantarum* has been associated with an improvement in the absorption of dietary proteins under inflammatory conditions [67]. However, in the same model of spontaneous colitis (IL-10 knockout mice), *L. plantarum* treatment decreases the expression and transport activity of PepT1 in the colon [68]. Importantly, PepT1 regulation has been shown for interventions administering viable probiotics. To our knowledge, whether these effects are mediated by EVs is unknown. Given the key role of bacterial EVs in mediating probiotic effects, it is likely that the regulation of PepT1 by *L. plantarum* or other probiotics is also mediated by extracellular vesicles.

This study provides new insights into the molecular mechanisms triggered by EVs from the probiotic EcN to ameliorate inflammation in intestinal epithelial cells. The main finding is the EV-mediated upregulation of miR-193a-3p and the accompanying downregulation of PepT1 protein. Importantly, this regulatory effect is only activated in epithelial cells exposed to inflammatory mediators. By this mechanism, EcN EVs may reduce inflammation in response to microbiota in IBD and other chronic inflammatory disorders by limiting the entrance of bacterial proinflammatory peptides. Moreover, downregulation of miR-193a-3p in IBD is related with the promotion of colitis-associated colorectal cancer [42,69] acting on the target mRNAs of the Interleukin-17 receptor D (IL-17RD) and the plasminogen activator urokinase (PLAU). IL-17RD is a multifunctional regulator of immune receptors and inflammatory signaling pathways [70], whereas PLAU is involved in cancer cell migration and angiogenesis [69]. In this context, upregulation of miR-193a-3p by EcN EVs may have additional beneficial effects preventing cancer onset and progression in chronically inflamed colon.

## 5. Conclusions

This study provides new insights into the regulation of PepT1 by EVs of the probiotic EcN in colonic epithelial cells under inflammatory conditions, emphasizing their potential use as a postbiotic strategy for treating IBD and associated cancer progression. Upregulation of miR-193a-3p and the subsequent downregulation of PepT1 in response to EcN EVs serve to limit the colonic uptake of bacterial proinflammatory peptides, thereby reducing intestinal inflammation in response to microbiota in chronic intestinal inflammation disorders. To the best of our knowledge, this is the first study providing evidence that EVs from a probiotic strain regulate PepT1 expression through upregulation of miR-193a-3p. Further studies in IBD animal models are needed to demonstrate the regulation of PepT1 and miR-193a-3p by EcN EVs in vivo.

IBD is a group of multifactorial diseases influenced by multiple mechanisms. The results presented here, together with previous findings of our group, show the potential of EcN EVs in (i) repairing and reinforcing the intestinal epithelial barrier through regulation of TFF3/miR-7-5p and tight junction proteins; (ii) reducing serotonin levels through regulation of SERT expression at both the transcriptional and posttranscriptional levels; and (iii) counteracting oxidative stress through differential regulation of pro-inflammatory and antioxidant enzymes. These findings strongly support the potential application of EVs from the probiotic EcN as a safe postbiotic strategy for IBD, acting at multiple levels to improve intestinal homeostasis. Clinical trials are necessary to confirm the benefits of interventions involving EcN EVs in leaky gut and chronic inflammatory conditions in humans.

## Data Availability

The original contributions presented in the study are included in the article/Supplementary Material, further inquiries can be directed to the corresponding authors.

## Supplementary Materials

The following supporting information can be downloaded at: https://www.mdpi.com/article/10.3390/nu16162719/s1. Figure S1: Cryo-TEM images of EVs isolated from EcN and ECOR12. Figure S2: Expression levels of the reference genes used for RT-qPCR assays.

## References

[1] Viennois E., Pujada A., Zen J., Merlin D. (2018). Function, regulation, and pathophysiological relevance of the POT superfamily, specifically PepT1 in inflammatory bowel disease. Compr. Physiol..

[2] Merlin D., Si-Tahar M., Sitaraman S.V., Eastburn K., Williams I., Liu X., Hediger M.A., Madara J.L. (2001). Colonic epithelial hPepT1 expression occurs in inflammatory bowel disease: Transport of bacterial peptides influences expression of MHC class 1 molecules. Gastroenterology.

[3] Geillinger K.E., Kipp A.P., Schink K., Röder P.V., Spanier B., Daniel H. (2014). Nrf2 regulates the expression of the peptide transporter PEPT1 in the human colon carcinoma cell line Caco-2. Biochim. Biophys. Acta Gen. Subj..

[4] Spanier B., Rohm F. (2018). Proton coupled oligopeptide transporter 1 (PepT1) function, regulation, and influence on the intestinal homeostasis. Compr. Physiol..

[5] Ingersoll S.A., Ayyadurai S., Charania M.A., Laroui H., Yan Y., Merlin D. (2012). The role and pathophysiological relevance of membrane transporter pept1 in intestinal inflammation and inflammatory bowel disease. Am. J. Physiol. Gastrointest. Liver Physiol..

[6] Viennois E., Ingersoll S.A., Ayyadurai S., Zhao Y., Wang L., Zhang M., Han M.K., Garg P., Xiao B., Merlin D. (2016). Critical Role of PepT1 in Promoting Colitis-Associated Cancer and Therapeutic Benefits of the Anti-inflammatory PepT1-Mediated Tripeptide KPV in a Murine Model. Cell. Mol. Gastroenterol. Hepatol..

[7] Viennois E., Pujada A., Sung J., Yang C., Gewirtz A.T., Chassaing B., Merlin D. (2020). Impact of PepT1 deletion on microbiota composition and colitis requires multiple generations. NPJ Biofilms Microbiomes.

[8] Newstead S. (2017). Recent advances in understanding proton coupled peptide transport via the POT family. Curr. Opin. Struct. Biol..

[9] Zhang Y., Viennois E., Zhang M., Xiao B., Han M.K., Walter L., Garg P., Merlin D. (2016). PepT1 Expression Helps Maintain Intestinal Homeostasis by Mediating the Differential Expression of miRNAs along the Crypt-Villus Axis. Sci. Rep..

[10] Wang C.Y., Liu S., Xie X.N., Tan Z.R. (2017). Regulation profile of the intestinal peptide transporter 1 (PepT1). Drug Des. Devel. Ther..

[11] Dalmasso G., Nguyen H.T., Charrier-Hisamuddin L., Yan Y., Laroui H., Demoulin B., Sitaraman S.V., Merlin D. (2010). PepT1 mediates transport of the proinflammatory bacterial tripeptide L-Ala-γ-D-Glu-meso-DAP in intestinal epithelial cells. Am. J. Physiol. Gastrointest. Liver Physiol..

[12] Laroui H., Yan Y., Narui Y., Ingersoll S.A., Ayyadurai S., Charania M.A., Zhou F., Wang B., Salaita K., Sitaraman S.V. (2011). L-Ala-γ-D-Glu-meso-diaminopimelic acid (DAP) interacts directly with leucine-rich region domain of nucleotide-binding oligomerization domain 1, increasing phosphorylation activity of receptor-interacting serine/threonine- protein kinase 2 and its interaction with nucleotide-binding oligomerization domain 1. J. Biol. Chem..

[13] Dalmasso G., Nguyen H.T., Ingersoll S.A., Ayyadurai S., Laroui H., Charania M.A., Yan Y., Sitaraman S.V., Merlin D. (2011). The PepT1-NOD2 signaling pathway aggravates induced colitis in mice. Gastroenterology.

[14] Caruso R., Warner N., Inohara N., Núñez G. (2014). NOD1 and NOD2: Signaling, host defense, and inflammatory disease. Immunity.

[15] Wuensch T., Ullrich S., Schulz S., Chamaillard M., Schaltenberg N., Rath E., Goebel U., Sartor R.B., Prager M., Büning C. (2014). Colonic expression of the peptide transporter PEPT1 is downregulated during intestinal inflammation and is not required for NOD2-dependent immune activation. Inflamm. Bowel. Dis..

[16] Dai X., Chen X., Chen Q., Shi L., Liang H., Zhou Z., Liu Q., Pang W., Hou D., Wang C. (2015). MicroRNA-193a-3p reduces intestinal inflammation in response to microbiota via down-regulation of colonic PepT1. J. Biol. Chem..

[17] Buyse M., Tsocas A., Walker F., Merlin D., Bado A. (2002). PepT1-mediated fMLP transport induces intestinal inflammation in vivo. Am. J. Physiol. Cell Physiol..

[18] Sonnenborn U., Schulze J. (2009). The non-pathogenic Escherichia coli strain Nissle 1917-features of a versatile probiotic. Microb. Ecol. Health Dis..

[19] Jacobi C.A., Malfertheiner P. (2011). *Escherichia coli* nissle 1917 (Mutaflor): New insights into an old probiotic bacterium. Dig. Dis..

[20] Behnsen J., Deriu E., Sassone-Corsi M., Raffatellu M. (2013). Probiotics: Properties, examples, and specific applications. Cold Spring Harb. Perspect. Biol..

[21] Ma L., Tu H., Chen T. (2023). Postbiotics in Human Health: A Narrative Review. Nutrients.

[22] Salminen S., Collado M.C., Endo A., Hill C., Lebeer S., Quigley E.M., Sanders M.E., Shamir R., Swann J.R., Szajewska H. (2021). The International Scientific Association of Probiotics and Prebiotics (ISAPP) consensus statement on the definition and scope of postbiotics. Nat. Rev. Gastroenterol. Hepatol..

[23] Olovo C.V., Wiredu Ocansey D.K., Ji Y., Huang X., Xu M. (2024). Bacterial membrane vesicles in the pathogenesis and treatment of inflammatory bowel disease. Gut Microbes.

[24] González-Lozano E., García-García J., Gálvez J., Hidalgo-García L., Rodríguez-Nogales A., Rodríguez-Cabezas M.E., Sánchez M. (2022). Novel Horizons in Postbiotics: Lactobacillaceae Extracellular Vesicles and Their Applications in Health and Disease. Nutrients.

[25] Chang C.J., Lin T.L., Tsai Y.L., Wu T.R., Lai W.F., Lu C.C., Lai H.C. (2019). Next generation probiotics in disease amelioration. J. Food Drug Anal..

[26] Díaz-Garrido N., Badia J., Baldomà L. (2021). Microbiota-derived extracellular vesicles in interkingdom communication in the gut. J. Extracell Vesicles.

[27] Alvarez C.S., Badia J., Bosch M., Giménez R., Baldomà L. (2016). Outer Membrane Vesicles and Soluble Factors Released by Probiotic Escherichia coli Nissle 1917 and Commensal ECOR63 Enhance Barrier Function by Regulating Expression of Tight Junction Proteins in Intestinal Epithelial Cells. Front. Microbiol..

[28] Diaz-Garrido N., Badia J., Baldomà L. (2022). Modulation of Dendritic Cells by Microbiota Extracellular Vesicles Influences the Cytokine Profile and Exosome Cargo. Nutrients.

[29] Cañas M.A., Fábrega M.J., Giménez R., Badia J., Baldomà L. (2018). Outer membrane vesicles from probiotic and commensal escherichia coli activate NOD1-mediated immune responses in intestinal epithelial cells. Front. Microbiol..

[30] Fábrega M.J., Rodríguez-Nogales A., Garrido-Mesa J., Algieri F., Badía J., Giménez R., Gálvez J., Baldomà L. (2017). Intestinal Anti-inflammatory Effects of Outer Membrane Vesicles from Escherichia coli Nissle 1917 in DSS-Experimental Colitis in Mice. Front. Microbiol..

[31] Martínez-Ruiz S., Olivo-Martínez Y., Cordero C., Rodríguez-Lagunas M.J., Pérez-Cano F.J., Badia J., Baldoma L. (2024). Microbiota-Derived Extracellular Vesicles as a Postbiotic Strategy to Alleviate Diarrhea and Enhance Immunity in Rotavirus-Infected Neonatal Rats. Int. J. Mol. Sci..

[32] Olivo-Martínez Y., Bosch M., Badia J., Baldomà L. (2023). Modulation of the Intestinal Barrier Integrity and Repair by Microbiota Extracellular Vesicles through the Differential Regulation of Trefoil Factor 3 in LS174T Goblet Cells. Nutrients.

[33] Olivo-Martínez Y., Martínez-Ruiz S., Cordero-Alday C., Bosch M., Badia J., Baldoma L. (2024). Modulation of Serotonin-Related Genes by Extracellular Vesicles of the Probiotic Escherichia coli Nissle 1917 in the Interleukin-1β-Induced Inflammation Model of Intestinal Epithelial Cells. Int. J. Mol. Sci..

[34] Ochman H., Selander R.K. (1984). Standard reference strains of Escherichia coli from natural populations. J. Bacteriol..

[35] Diaz-Garrido N., Fábrega M.J., Vera R., Giménez R., Badia J., Baldomà L. (2019). Membrane vesicles from the probiotic Nissle 1917 and gut resident Escherichia coli strains distinctly modulate human dendritic cells and subsequent T cell responses. J. Funct. Foods.

[36] Schindelin J., Arganda-Carreras I., Frise E., Kaynig V., Longair M., Pietzsch T., Preibisch S., Rueden C., Saalfeld S., Schmid B. (2012). Fiji: An open-source platform for biological-image analysis. Nat. Methods.

[37] Huang L.K., Wang M.J. (1995). Image thresholding by minimizing the measures of fuzziness. Pattern Recognit..

[38] Dalmasso G., Nguyen H.T., Yan Y., Laroui H., Charania M.A., Obertone T.S., Sitaraman S.V., Merlin D. (2011). MicroRNA-92b regulates expression of the oligopeptide transporter PepT1 in intestinal epithelial cells. Am. J. Physiol. Gastrointest. Liver Physiol..

[39] Buyse M., Charrier L., Sitaraman S., Gewirtz A., Merlin D. (2003). Interferon-γ Increases hPepT1-Mediated Uptake of Di-Tripeptides Including the Bacterial Tripeptide fMLP in Polarized Intestinal Epithelia. Am. J. Pathol..

[40] Vavricka S.R., Musch M.W., Fujiya M., Kles K., Chang L., Eloranta J.J., Kullak-Ublick G.A., Drabik K., Merlin D., Chang E.B. (2006). Tumor necrosis factor-α and interferon-γ increase PepT1 expression and activity in the human colon carcinoma cell line Caco-2/bbe and in mouse intestine. Pflugers Arch..

[41] Wang P., Lu Y.Q., Wen Y., Yu D.Y., Ge L., Dong W.R., Xiang L.X., Shao J.Z. (2013). IL-16 Induces Intestinal Inflammation via PepT1 Upregulation in a Pufferfish Model: New Insights into the Molecular Mechanism of Inflammatory Bowel Disease. J. Immunol..

[42] Berezin A.E., Poplyonkin E.I. (2020). Diagnostic and therapeutic value of micro-RNAs in inflammatory bowel disease. Biomed. Res. Ther..

[43] Ayyadurai S., Charania M.A., Xiao B., Viennois E., Merlin D. (2013). PepT1 expressed in immune cells has an important role in promoting the immune response during experimentally induced colitis. Lab. Investig..

[44] Ayyadurai S., Charania M.A., Xiao B., Viennois E., Zhang Y., Merlin D. (2014). Colonic miRNA expression/secretion, regulated by intestinal epithelial PepT1, plays an important role in cell-to-cell communication during colitis. PLoS ONE.

[45] Miyake M., Fujishima M., Nakai D. (2017). Inhibitory potency of marketed drugs for ulcerative colitis and Crohn’s disease on PEPT1. Biol. Pharm. Bull..

[46] Bruckmueller H., Martin P., Kähler M., Haenisch S., Ostrowski M., Drozdzik M., Siegmund W., Cascorbi I., Oswald S. (2017). Clinically Relevant Multidrug Transporters Are Regulated by microRNAs along the Human Intestine. Mol. Pharm..

[47] Cañas M.A., Giménez R., Fábrega M.J., Toloza L., Baldomà L., Badia J. (2016). Outer Membrane Vesicles from the Probiotic Escherichia coli Nissle 1917 and the Commensal ECOR12 Enter Intestinal Epithelial Cells via Clathrin-Dependent Endocytosis and Elicit Differential Effects on DNA Damage. PLoS ONE.

[48] Petrosino J.F. (2018). The microbiome in precision medicine: The way forward. Genome Med..

[49] Behrouzi A., Nafari A.H., Siadat S.D. (2019). The significance of microbiome in personalized medicine. Clin. Transl. Med..

[50] Kotla N.G., Rochev Y. (2023). IBD disease-modifying therapies: Insights from emerging therapeutics. Trends Mol. Med..

[51] Long A.G., Lundsmith E.T., Hamilton K.E. (2017). Inflammation and Colorectal Cancer. Curr. Colorectal. Cancer Rep..

[52] Pesce M., Seguella L., Del Re A., Lu J., Palenca I., Corpetti C., Rurgo S., Sanseverino W., Sarnelli G., Esposito G. (2022). Next-Generation Probiotics for Inflammatory Bowel Disease. Int. J. Mol. Sci..

[53] De Filippis F., Esposito A., Ercolini D. (2022). Outlook on next-generation probiotics from the human gut. Cell. Mol. Life Sci..

[54] Jia K., Tong X., Wang R., Song X. (2018). The clinical effects of probiotics for inflammatory bowel disease: A meta-analysis. Medicine.

[55] Martyniak A., Medyńska-Przęczek A., Wędrychowicz A., Skoczeń S., Tomasik P.J. (2021). Prebiotics, Probiotics, Synbiotics, Paraprobiotics and Postbiotic Compounds in IBD. Biomolecules.

[56] Mosca A., Abreu YAbreu A.T., Gwee K.A., Ianiro G., Tack J., Nguyen T.V., Hill C. (2022). The clinical evidence for postbiotics as microbial therapeutics. Gut Microbes.

[57] Wegh C.A.M., Geerlings S.Y., Knol J., Roeselers G., Belzer C. (2019). Postbiotics and Their Potential Applications in Early Life Nutrition and Beyond. Int. J. Mol. Sci..

[58] Afzaal M., Saeed F., Bibi M., Ejaz A., Shah Y.A., Faisal Z., Ateeq H., Akram N., Asghar A., Shah M.A. (2023). Nutritional, pharmaceutical, and functional aspects of rambutan in industrial perspective: An updated review. Food Sci. Nutr..

[59] Yang J., Shin T.-S., Kim J.S., Jee Y.-K., Kim Y.-K. (2022). A new horizon of precision medicine: Combination of the microbiome and extracellular vesicles. Exp. Mol. Med..

[60] Xie J., Li Q., Nie S. (2024). Bacterial extracellular vesicles: An emerging postbiotic. Trends Food Sci. Technol..

[61] Krzyżek P., Marinacci B., Vitale I., Grande R. (2023). Extracellular Vesicles of Probiotics: Shedding Light on the Biological Activity and Future Applications. Pharmaceutics.

[62] Shen Q., Huang Z., Yao J., Jin Y. (2022). Extracellular vesicles-mediated interaction within intestinal microenvironment in inflammatory bowel disease. J. Adv. Res..

[63] Sultan S., Mottawea W., Yeo J., Hammami R. (2021). Gut Microbiota Extracellular Vesicles as Signaling Molecules Mediating Host-Microbiota Communications. Int. J. Mol. Sci..

[64] Kang C.S., Ban M., Choi E.J., Moon H.G., Jeon J.S., Kim D.K., Park S.K., Jeon S.G., Roh T.Y., Myung S.J. (2013). Extracellular Vesicles Derived from Gut Microbiota, Especially Akkermansia muciniphila, Protect the Progression of Dextran Sulfate Sodium-Induced Colitis. PLoS ONE.

[65] Ma L., Shen Q., Lyu W., Lv L., Wang W., Yu M., Yang H., Tao S., Xiao Y. (2022). Clostridium butyricum and Its Derived Extracellular Vesicles Modulate Gut Homeostasis and Ameliorate Acute Experimental Colitis. Microbiol. Spectr..

[66] Molina-Tijeras J.A., Gálvez J., Rodríguez-Cabezas M.E. (2019). The immunomodulatory properties of extracellular vesicles derived from probiotics: A novel approach for the management of gastrointestinal diseases. Nutrients.

[67] Chen H.Q., Shen T.Y., Zhou Y.K., Zhang M., Chu Z.X., Hang X.M., Qin H.L. (2010). Lactobacillus plantarum consumption increases PepT1-mediated amino acid absorption by enhancing protein kinase C activity in spontaneously colitic mice. J. Nutr..

[68] Chen H.Q., Yang J., Zhang M., Zhou Y.K., Shen T.Y., Chu Z.X., Zhang M., Hang X.M., Jiang Y.Q., Qin H.L. (2010). Lactobacillus plantarum ameliorates colonic epithelial barrier dysfunction by modulating the apical junctional complex and PepT1 in IL-10 knockout mice. Am. J. Physiol. Gastrointest. Liver Physiol..

[69] Lin M., Zhang Z., Gao M., Yu H., Sheng H., Huang J. (2019). MicroRNA-193a-3p suppresses the colorectal cancer cell proliferation and progression through downregulating the PLAU expression. Cancer Manag. Res..

[70] Pande S., Yang X., Friesel R. (2021). Interleukin-17 receptor D (Sef) is a multi-functional regulator of cell signaling. Cell Commun. Signal..

