# Supplementary material for: Extracellular Vesicles of the Probiotic Escherichia coli Nissle 1917 Reduce PepT1 Levels in IL-1β-Treated Caco-2 Cells via Upregulation of miR-193a-3p
Source: Nutrients. 2024 Aug 15;16(16):2719. doi: 10.3390/nu16162719 (PMC11356789; doi:10.3390/nu16162719)
Supplement: Supplementary file 1 [file nutrients-16-02719-s001.zip › nutrients-3136551-supplementary Figure S1.pdf]

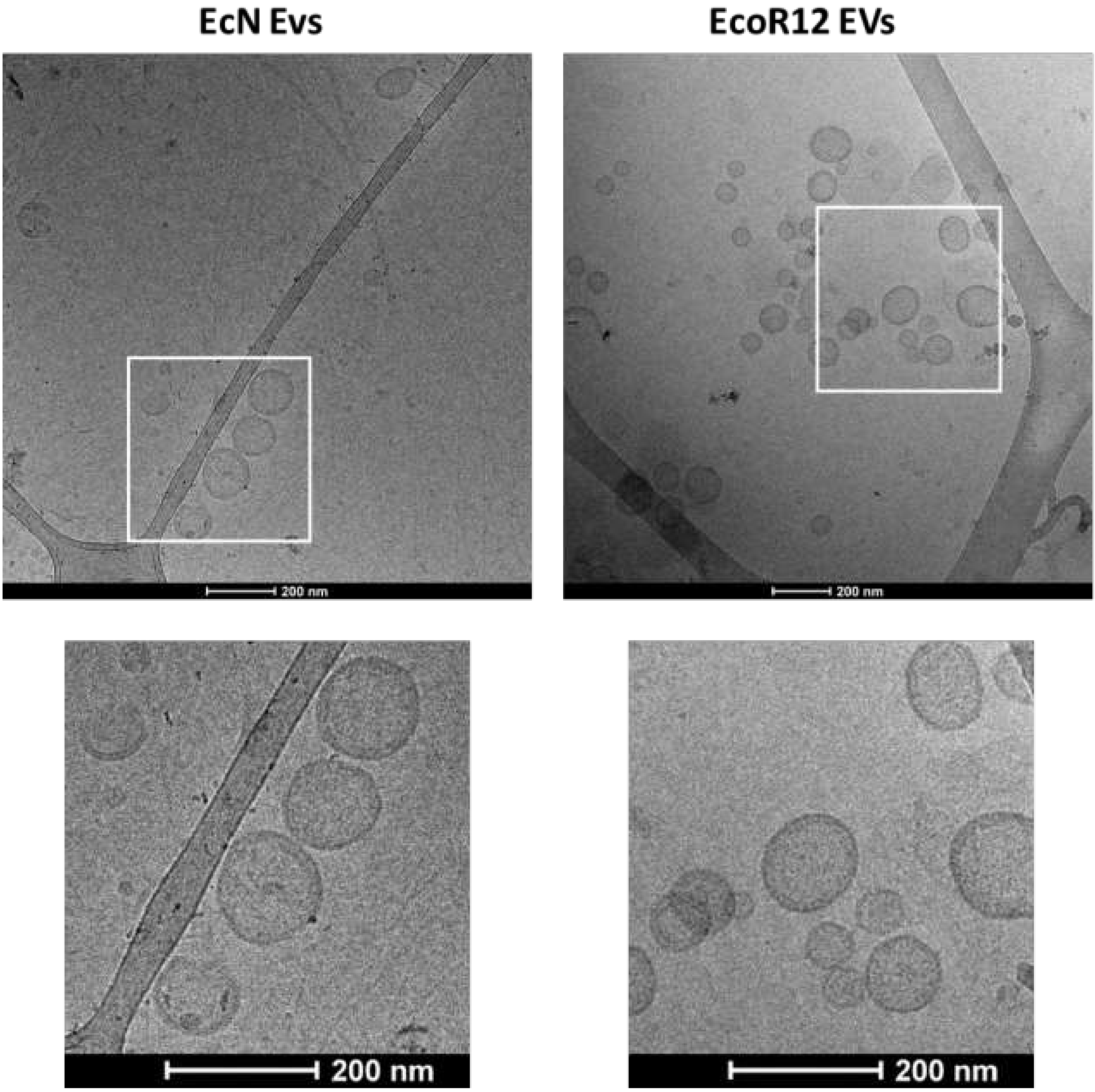

Figure S1. Cryo-TEM images of the EVs isolated from EcN and ECOR12. Representative images of plungefrozen EVs are shown. Analysis was performed with fresh EVs samples resuspended in 0.1 M phosphate buffer (pH 7.2) as described previously [35]. Briefly, one drop of each EV suspension (5 l) was applied on the carbon surface of a glow-discharged Lacey Carbon 300 mesh copper grid (Ted Pella, USA). The sample was allowing to adsorb for 4 min at 100% humidity inside the chamber of the Vitrobot Mark III (FEI Company, Eindhoven, Netherlands). The excess of liquid was automatically blotted with filter paper, followed by cryo-immobilization by plunge freezing in liquefied ethane. The vitrified sample was stored in liquid nitrogen until its observation in the cryo-electron microscope. Plunge-frozen samples were transferred to a Tecnai F20 EM (FEI, Eindhoven, The Netherlands) using a cryo-holder system (Gatan, Pleasanton, USA). The sample was examined at 200 kV, at a temperature ranging from 179 tq 170°C using low-dose imaging conditions. Low-dose images were recorded with a 4096 × 4096-pixel CCD Eagle camera (FEI, Eindhoven, The Netherlands).
