# Supplementary material for: Extracellular Vesicles of the Probiotic Escherichia coli Nissle 1917 Reduce PepT1 Levels in IL-1β-Treated Caco-2 Cells via Upregulation of miR-193a-3p
Source: Nutrients. 2024 Aug 15;16(16):2719. doi: 10.3390/nu16162719 (PMC11356789; doi:10.3390/nu16162719)
Supplement: Supplementary file 1 [file nutrients-16-02719-s001.zip › nutrients-3136551-supplementary Figure S2.pdf]

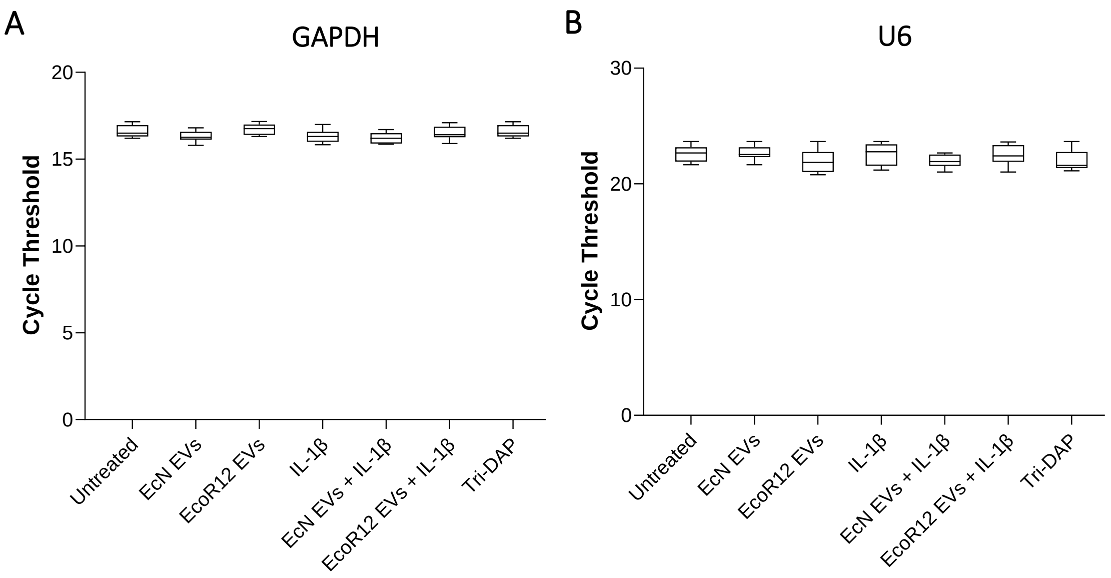

Figure S2: Expression levels of the reference genes used for RT-qPCR assays. The cycle threshold (Ct) values for GAPDH and U6 in Caco-2 cells treated under indicated experimental conditions are shown. Data are expressed as mean ± SEM (n = 3 independent biological replicates). Statistical differences were assessed with one-way ANOVA, followed by post hoc Tukey's. The variability in Ct values for GAPDH and U6 was less than 1 cycle across the different experimental conditions, confirming their suitability as reference genes for gene expression normalization in this study.
